# Hsp90 Chaperones Bluetongue Virus Proteins and Prevents Proteasomal Degradation

**DOI:** 10.1128/JVI.00898-19

**Published:** 2019-09-30

**Authors:** Bjorn-Patrick Mohl, Polly Roy

**Affiliations:** a Department of Pathogen Molecular Biology, Faculty of Infectious and Tropical Diseases, London School of Hygiene and Tropical Medicine, London, United Kingdom; Loyola University Chicago

**Keywords:** bluetongue virus, Hsp70, Hsp90, proteasome

## Abstract

The molecular chaperone machinery is important for the maintenance of protein homeostasis within the cells. The principle activities of the chaperone machinery are to facilitate protein folding and organize conformationally dynamic client proteins. Prominent among the members of the chaperone family are heat shock protein 70 (Hsp70) and 90 (Hsp90). Like cellular proteins, viral proteins depend upon molecular chaperones to mediate their stabilization and folding. Bluetongue virus (BTV), which is a model system for the *Reoviridae* family, is a nonenveloped arbovirus that causes hemorrhagic disease in ruminants. This constitutes a significant burden upon animals of commercial significance, such as sheep and cattle. Here, for the first time, we examined the role of chaperone proteins in the viral lifecycle of BTV. Using a combination of molecular, biochemical, and microscopic techniques, we examined the function of Hsp90 and its relevance to BTV replication. We demonstrate that Hsp70, the chaperone that is commonly usurped by viral proteins, does not influence virus replication, while Hsp90 activity is important for virus replication by stabilizing BTV proteins and preventing their degradation via the ubiquitin-proteasome pathway. To our knowledge this is the first report showing the involvement of Hsp90 as a modulator of BTV infection.

**IMPORTANCE** Protein chaperones are instrumental for maintaining protein homeostasis, enabling correct protein folding and organization; prominent members include heat shock proteins 70 and 90. Virus infections place a large burden on this homeostasis. Identifying and understanding the underlying mechanisms that facilitate Bluetongue virus replication and spread through the usurpation of host factors is of primary importance for the development of intervention strategies. Our data identify and show that heat shock protein 90, but not heat shock protein 70, stabilizes bluetongue virus proteins, safeguarding them from proteasomal degradation.

## Abstract (precis)

Protein chaperones are instrumental for maintaining protein homeostasis, enabling correct protein folding and organization; prominent members include heat shock proteins 70 and 90. Virus infections place a large burden on this homeostasis. Identifying and understanding the underlying mechanisms that facilitate Bluetongue virus replication and spread through the usurpation of host factors is of primary importance for the development of intervention strategies. Our data identify and show that heat shock protein 90, but not heat shock protein 70, stabilizes bluetongue virus proteins, safeguarding them from proteasomal degradation.

## INTRODUCTION

Molecular chaperones are pivotal actors in the process of protein homeostasis, which orchestrates the harmonization of protein synthesis, folding, trafficking, assembly, and turnover by facilitating protein folding, disaggregation, and degradation (1). In this process, newly synthesized proteins are supported by chaperones in adopting their biologically active conformations (2). Two of the most prominent chaperone family members are heat shock protein 70 (Hsp70) and Hsp90. While Hsp70 is monomeric and Hsp90 is homodimeric, both utilize ATPase activity that drive conformational changes to facilitate client protein binding, stabilization, and release (3–6). Furthermore, both function in concert with cofactors to facilitate protein homeostasis (1). Hsp90 is an evolutionarily highly conserved and abundant protein, constituting up to 2% of total cellular proteins that can increase to 6% in stressed conditions (7). Each monomer is comprised of three domains: an N-terminal ATP-binding domain, a middle domain that regulates the ATPase activity of the N-terminal domain, and a C-terminal dimerization domain (4). The Hsp90 homodimer conformational cycle involves ATP binding at the N-terminal ATPase domain, mediating the closing of the dimer and that reopens following ATP hydrolysis (6, 8). An inability of Hsp90 to successfully chaperone its client proteins directs these into proteasomal degradation via the ubiquitin-proteasome pathway (9–11). Given the importance of Hsp90 in mediating protein homeostasis, accumulating evidence highlights the importance of this cellular protein to virus replication, through either a direct or indirect reliance upon it (12).

Bluetongue virus (BTV), the prototype member of the *Orbivirus* genus in the *Reoviridae* family, is an insect-vectored emerging pathogen causing hemorrhagic disease in wild ruminants and livestock (with mortality reaching 70% in sheep) in many parts of the world. BTV is an icosahedral double-capsid virus with an architecturally complex structure. The double-capsid is comprised of 7 structural proteins (VP1 to VP7) that are organized in two concentric protein shells encasing a genome of 10 segmented double-stranded RNAs (dsRNAs) (13–15). In addition to the seven structural proteins, four nonstructural proteins (NS1 to NS4) are also synthesized in the infected host cells (16, 17). BTV also utilizes a number of essential host factors to facilitate successful infection, replication, and viral spread. These include, but are not limited to, the late endosome-specific lipid lysobisphosphatidic acid (18), casein kinase 2 (19, 20), calpactin (21), and the NEDD4 family of proteins (22), respectively. BTV has also been found to require multi vesicular body components and exocytic pathway proteins for infectious virus production (22).

In a recent study of the phosphoproteome of BTV-infected cells, our data predicted the potential novel importance of Hsp90AB1, here referred to as Hsp90, activity in BTV-infected cells, but not that of Hsp70 (23). We consequently set out to validate this prediction and elucidate the role of Hsp90 during BTV infection. Here, we performed a series of studies, using a combination of molecular, biochemical, and microscopic techniques. Using specific pharmacological inhibitors and siRNA knockdowns, we demonstrate that Hsp90 activity is required for BTV replication. This may enable the development of novel therapeutic interventions during acute infections to mitigate disease progression.

## RESULTS

### Inhibition of Hsp90 decreases viral protein levels in BTV-infected cells.

**FIG 1:**
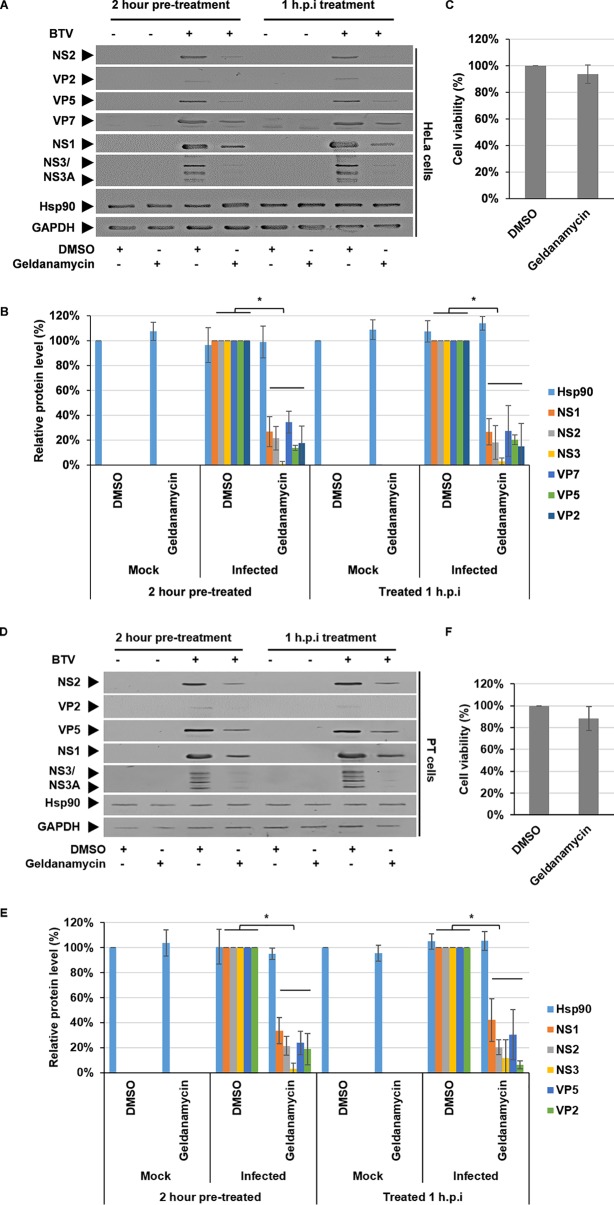

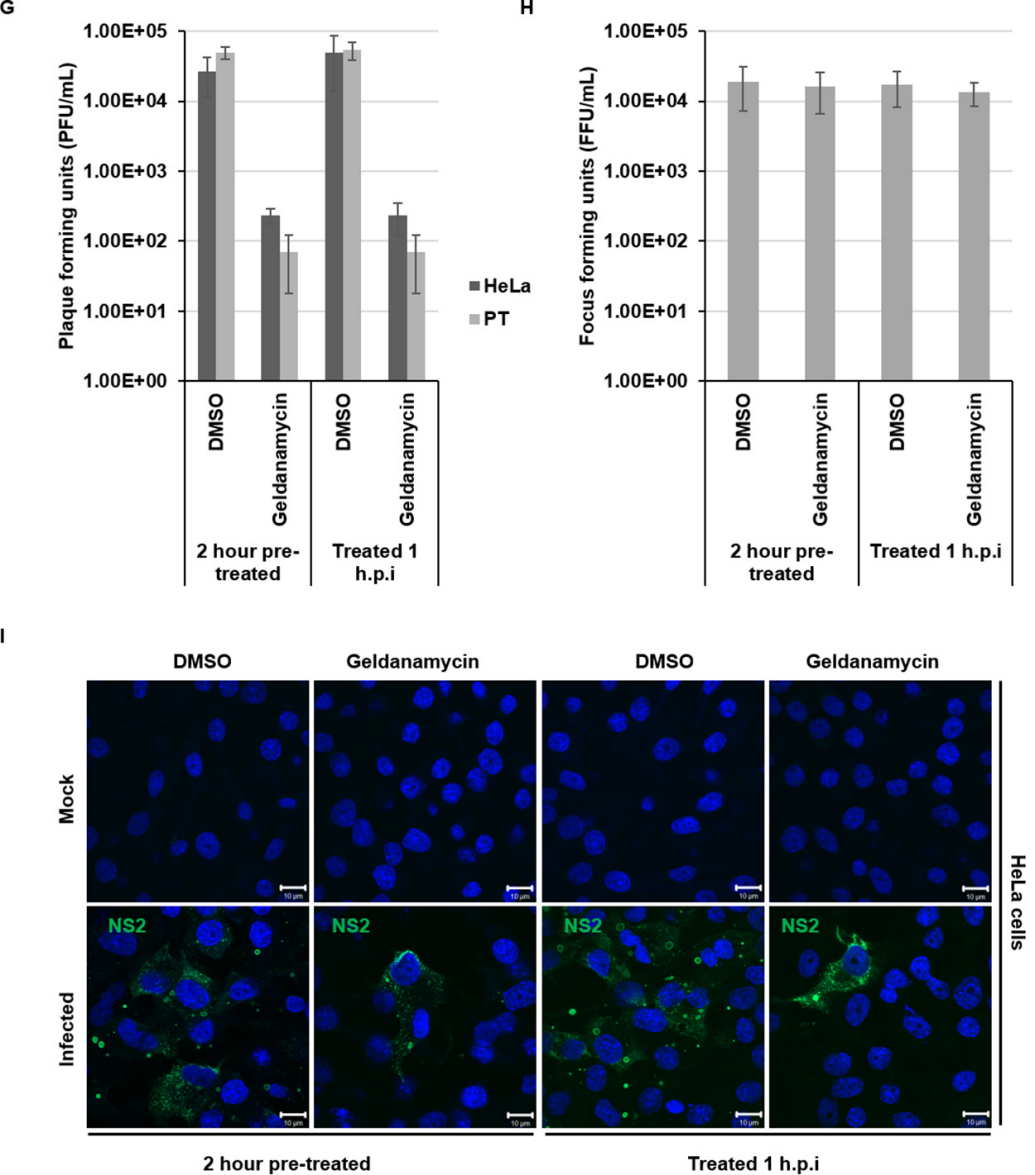
Inhibition of Hsp90 activity decreases viral protein levels in BTV-infected cells. HeLa cells (A to C) and sheep PT cells (D to F) were treated with DMSO or 300 nM geldanamycin for 2 h prior to or at 1 hpi with BTV1 (MOI = 1) and then harvested at 18 hpi .As controls, mock-infected and DMSO-treated cells were included. (A and D) Samples were analyzed by Western blotting with specific antibodies as indicated. (B and E) Densitometry analysis results for the Western blots are expressed as percentages, normalized to GAPDH. (C and F) Viability of cells treated with DMSO or 300 nM geldanamycin for 24 h. (G) Virus titers (PFU/ml) derived from HeLa and sheep PT cells treated as described above. (H) Focus-forming units (FFU)/ml at 18 hpi using HeLa cells infected with BTV1 (MOI = 1) and treated as described above. (I) Immunofluorescence microscopy of HeLa cells and virus inclusion bodies denoted by NS2 staining depicts infected cells. White bars, 10 μm. Error bars represent the SD values of stimulations from three independent experiments. An asterisk (*) denotes a significant difference from the control (*P* < 0.05).

In order to obtain direct evidence that Hsp90 activity is involved during the virus life cycle, we used a specific inhibitor for Hsp90, geldanamycin, which binds the N-terminal ATP/ADP-binding domain of Hsp90 and interferes with its ability to stabilize client proteins (6). HeLa cells were either pretreated 2 h prior to infection or 1 h postinfection (hpi) with dimethyl sulfoxide (DMSO) or 300 nM geldanamycin and infected at a multiplicity of infection (MOI) of 1. At 18 hpi, replicate samples were harvested for Western blot analysis and virus titer determination. Lysates were then analyzed by Western blotting using specific antibodies, which showed comparable decreases in both structural (VP2, VP5, and VP7) and nonstructural (NS1, NS2, and NS3) protein levels, while Hsp90 protein levels remained unperturbed by the inhibition (Fig. 1A). Densitometry quantification confirmed significant decreases in protein levels between 70 and 95%; specifically, NS1 decreased ∼70% ± 10%, NS2 decreased ∼80% ± 11%, NS3 decreased ∼95% ± 2%, VP2 decreased ∼85% ± 15%, VP5 decreased ∼85% ± 5%, and VP7 decreased ∼70% ± 15%, normalized to GAPDH (glyceraldehyde-3-phosphate dehydrogenase), under both experimental conditions (Fig. 1B). To address whether the inhibitor treatment decreased viral protein levels due to cellular toxicity, the effect of the inhibitor on cell viability was assessed by an MTT [3-(4,5-dimethylthiazol-2-yl)-2,5-diphenyltetrazolium bromide] assay. The data showed that 300 nM geldanamycin was not toxic to the HeLa cells at this concentration (Fig. 1C). Moreover, we also observed an ∼2-log_10_ decrease in virus titer under both conditions (Fig. 1G). Cumulatively, these data suggest that Hsp90 is required for viral replication after cell entry.

Whether the BTV infection in natural host cells requires the same cellular chaperone, we subsequently extended our study using sheep cells (PT cells), derived from a natural host species. As described for HeLa cells, the same experimental conditions were applied to sheep PT cells. At 18 hpi, Western blot analysis of infected cell lysates showed comparable decreases of both structural (VP2 and VP5) and nonstructural (NS1, NS2, and NS3) protein levels, whereas Hsp90 protein levels remained unperturbed by the inhibition (Fig. 1D). Similar to the HeLa cell infections, NS1 decreased ∼65% ± 12%, NS2 decreased ∼80% ± 5%, NS3 decreased ∼90% ± 5%, VP2 decreased ∼85% ± 10%, and VP5 decreased ∼75% ± 15% (Fig. 1E). As had been done for HeLa cells, we examined whether the inhibitor treatment decreased viral protein levels in PT cells due to cellular toxicity; cell viability was assessed by an MTT assay. The data showed that 300 nM geldanamycin was not toxic to the PT cells at this concentration (Fig. 1F). Further, as observed in HeLa cells, we also observed an ∼2-log_10_ decrease under both conditions (Fig. 1G). Cumulatively, there appeared to be no significant difference in the examined protein levels nor virus titers between samples treated prior to infection or postinfection. To further address whether the decrease in viral protein levels was due to a difference in the percentage of cells successfully infected in the DMSO and geldanamycin-treated samples, we conducted a focus-forming assay and confocal microscopy. HeLa cells were treated as described above and fixed 18 hpi for the focus-forming assay to determine the focus-forming units (FFU)/ml and indirect immunofluorescence using the NS2 antibody as a marker for infection. Determination of the FFU/ml showed no difference between DMSO and geldanamycin-treated samples (Fig. 1H). Furthermore, confocal microscopy confirmed the successful infection of the cells under both conditions (Fig. 1I). These data indicate that Hsp90 inhibition affected the viral life cycle after virus entry, across different cell lines, in both HeLa cells (Fig. 1A, B, C, G, H, and I) and sheep PT cells (Fig. 1D, E, F, and G).

**FIG 2:**
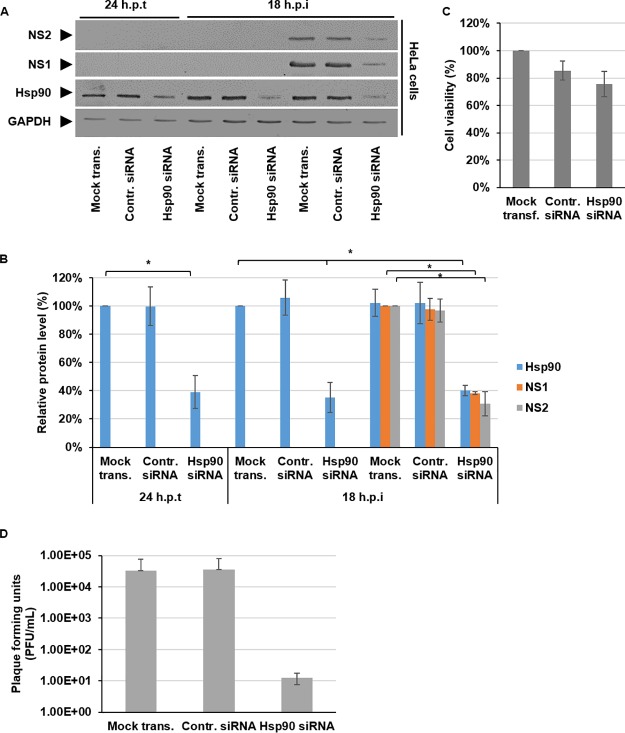
siRNA knockdown of Hsp90 decreases BTV protein levels. HeLa cells were either mock transfected or transfected with 400 nM control siRNA or Hsp90 siRNA for 24 h. Transfected cells were then infected with BTV1 (MOI = 1) for 18 h. (A) Samples were analyzed by Western blotting with specific antibodies as indicated. (B) Densitometry analysis results for the Western blots are expressed as percentages representing the NS1, NS2, and Hsp90 protein levels, normalized to GAPDH. (C) Viability of cells transfected with mock, negative-control siRNA, and Hsp90 siRNA for 42 h. (D) Virus titers (PFU/ml) derived from HeLa cells at 18 hpi. Error bars represent the SD values of stimulations from three independent experiments. An asterisk (*) denotes a significant difference from the control (*P* < 0.05).

To support the data obtained using the Hsp90 inhibitor and mitigate the possibility of off-target effects, we subsequently undertook small interfering RNA (siRNA) knockdown experiments targeting Hsp90. HeLa cells were mock transfected or transfected either with a control siRNA or with Hsp90 siRNA for 24 h prior to infection. At 24 h posttransfection (hpt), the samples were assessed to confirm the successful knockdown of Hsp90 protein levels prior to infection. Cells were then infected with BTV, harvested 18 hpi, and analyzed by Western blotting (Fig. 2A). Densitometry quantification confirmed that at the time of infection (24 hpt), Hsp90 protein levels normalized to GAPDH had decreased by ∼60% ± 10%. Subsequent to infection, we observed a decrease in NS1 and NS2 protein levels that correlated to the corresponding decreased levels of Hsp90. The level Hsp90 protein at 18 hpi was decreased by ∼60% ± 4%. This decrease correlated with an ∼60% ± 12% decrease in NS1 protein levels and an ∼70% ± 8% decrease in NS2 protein level (Fig. 2B). To address whether the siRNA knockdown decreased viral protein levels due to cellular toxicity, the effect of the knockdown on cell viability was assessed by using an MTT assay. The data showed that HeLa cell viability decreased ∼24% ± 9% using the Hsp90 siRNA; however, this decrease was comparable to the decrease observed using the negative-control siRNA, which decreased cell viability by ∼15% ± 7% (Fig. 2C). Furthermore, when supernatants from Hsp90 siRNA knockdown samples were analyzed, we observed a decrease in virus titer of ∼3 log_10_ (Fig. 2D). The decreases in viral protein levels and virus titers observed using siRNA knockdown of Hsp90 correlate with the decreases observed using the chemical Hsp90 inhibitor.

**FIG 3:**
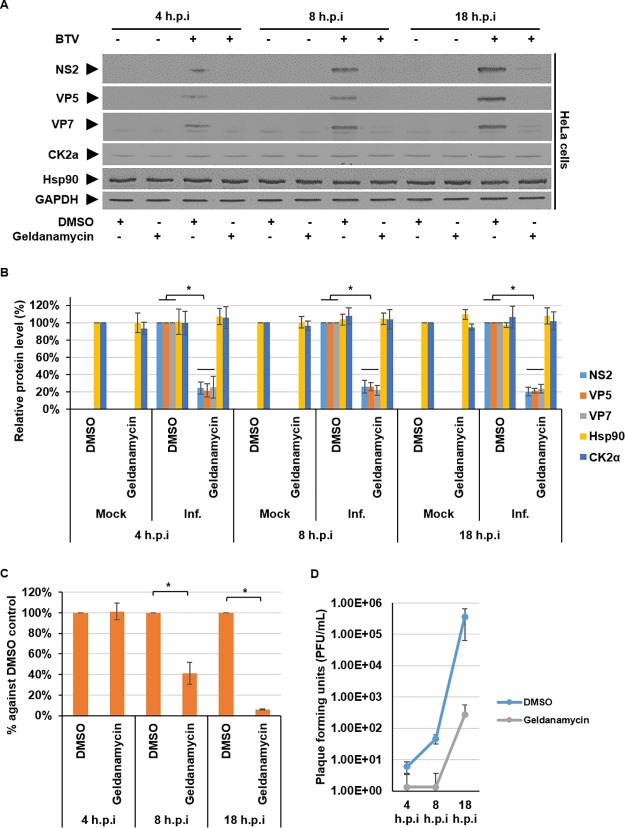
Inhibition of Hsp90 activity during an infection time course decreases viral replication. HeLa cells were mock infected or infected with BTV1 (MOI = 1) and treated with DMSO or 300 nM geldanamycin at 1 hpi. The cells were harvested 4, 8, and 18 hpi. (A) Samples were analyzed by Western blotting, using specific antibodies as indicated. (B) Densitometry analysis results for the Western blots are expressed as percentages representing NS2, VP5, VP7, Hsp90, and CK2α protein levels, normalized to GAPDH. (C) Viral genomic copy numbers using segment S6 were quantified by qRT-PCR. Quantities of S6 in geldanamycin-treated samples were compared to the DMSO-treated control in the same experiment. (D) Virus titers (PFU/ml) derived from HeLa cells treated as described above. Error bars represent the SD values of stimulations from three independent experiments. An asterisk (*) denotes a significant difference from the control (*P* < 0.05).

To further elucidate how inhibition of Hsp90 by geldanamycin was affecting virus replication, we examined viral genome and protein synthesis alongside viral particle assembly during BTV infection at 4, 8, and 18 hpi Concurrently, we monitored the host cell protein CK2α, which forms a complex with Hsp90 (24, 25) and is also essential for BTV virus replication (20). HeLa cells were mock infected or infected with BTV (MOI = 1) and treated with DMSO or 300 nM geldanamycin at 1 hpi. Lysates were then analyzed by Western blotting using specific antibodies, which showed comparable decreases of both structural (VP5 and VP7) and nonstructural (NS2) protein levels, while host cell Hsp90 and CK2α protein levels remained unperturbed by the inhibition (Fig. 3A). Densitometry quantification confirmed significant decreases in viral protein levels at all examined time points. Specifically, at 4 hpi NS2 decreased ∼75% ± 7%, VP5 decreased ∼79% ± 8%, and VP7 decreased ∼75% ± 12%. At 8 hpi NS2 decreased ∼74% ± 7%, VP5 decreased ∼74% ± 5%, and VP7 decreased ∼78% ± 5%, and at 18 hpi NS2 decreased ∼80% ± 5%, VP5 decreased ∼79% ± 3%, and VP7 decreased ∼76% ± 5%, normalized to GAPDH (Fig. 3B). To assay dsRNA synthesis, we quantified viral genome copy numbers using quantitative reverse transcription-PCR (qRT-PCR). Samples were treated and harvested as described above. Viral RNA was isolated and, using segment 6 as a genome representative, quantified. The qRT-PCR comparison results of DMSO-treated and geldanamycin-treated samples showed that at 4 hpi there was no significant decrease in genome copy numbers. However, 8 hpi the genome copy numbers had decreased ∼60% ± 10%, and at 18 hpi the genome copy numbers had decreased ∼94% ± 6% (Fig. 3C). Furthermore, when supernatants of samples were analyzed, the kinetics of virus production showed that geldanamycin treatment decreased the virus titer at 8 and 18 hpi by ∼2 to 3 log_10_ compared to the DMSO-treated control (Fig. 3D).

### Interaction of Hsp90 and BTV proteins.

**FIG 4:**
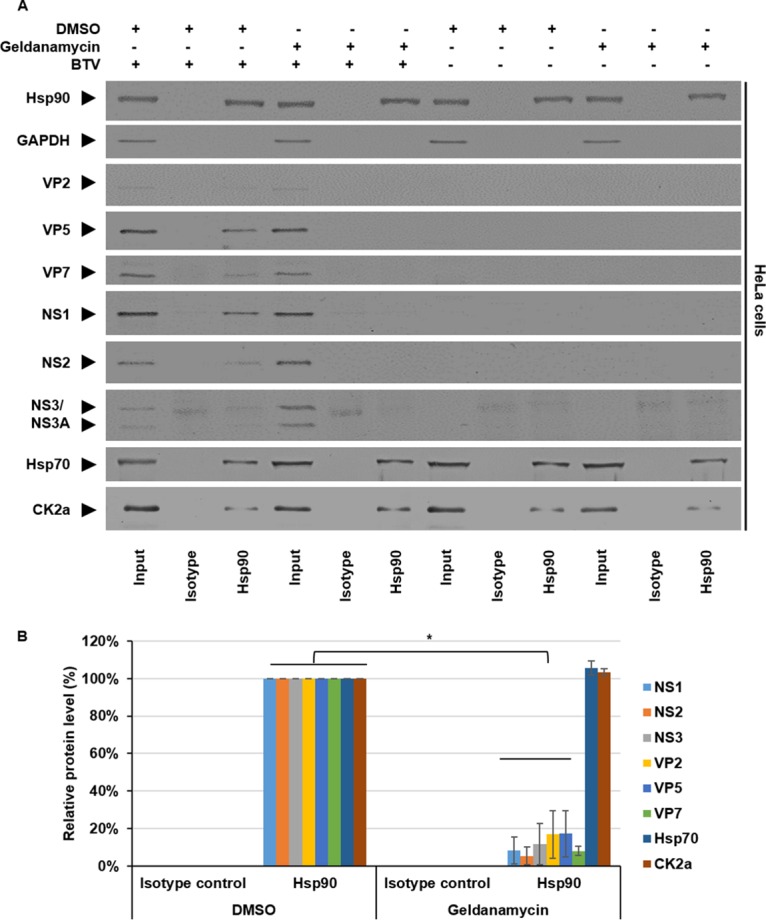
Hsp90 coimmunoprecipitation analysis of BTV proteins. HeLa cells were mock infected or infected with BTV1 (MOI = 1), and at 17 hpi the cells were treated with DMSO or 300 nM geldanamycin for 1 h. The cells were harvested 18 hpi in the presence of DMSO or 300 nM geldanamycin. A Co-IP assay was performed with an anti-Hsp90 antibody (11405-1-AP). (A) Samples were analyzed by Western blotting with specific antibodies as indicated for coimmunoprecipitating BTV proteins. (B) Densitometry analysis results for Western blotting are expressed as percentages representing NS1, NS2, NS3, VP2, VP5, VP7, Hsp70, and CK2α protein levels, normalized to Hsp90. Error bars represent the SD values of stimulations from three independent experiments. An asterisk (*) denotes a significant difference from the control (*P* < 0.05).

To investigate the interaction between Hsp90 and BTV proteins, coimmunoprecipitation (Co-IP) assays were performed. To examine the interaction of BTV proteins with Hsp90, HeLa cells were either mock infected or infected with BTV, and at 17 hpi the cells were treated with DMSO or 300 nM geldanamycin for 1 h. Cells were harvested 18 hpi in the presence of DMSO or 300 nM geldanamycin. Cell lysates were incubated with protein A-Sepharose beads conjugated with an isotype control or anti-Hsp90 antibody. The protein complexes coimmunoprecipitated with the beads were eluted and analyzed by Western blotting with antibodies against Hsp90, VP2, VP5, VP7, NS1, NS2, NS3, Hsp70, CK2α, and GAPDH (Fig. 4A). Alongside Hsp90, coimmunoprecipitating bands were detected for VP2, VP5, VP7, NS1, NS2, NS3, Hsp70, and CK2α. No GAPDH was detectable in the immunoprecipitated samples. Samples derived from cells that had been treated for 1 h with 300 nM geldanamycin showed significantly lower quantities of coimmunoprecipitating bands. Densitometry quantification confirmed significant decreases of viral protein levels between 80 and 95%, specifically, NS1 decreased ∼92% ± 7%, NS2 decreased ∼95% ± 4%, NS3 decreased ∼89% ± 10%, VP2 decreased ∼83% ± 12%, VP5 decreased ∼83% ± 12%, and VP7 decreased ∼92% ± 3%, normalized to the Hsp90 eluted from the beads (Fig. 4B). There was no significant decrease in Hsp70 and CK2α host protein levels, indicating that the Hsp90 chaperone complexes remained intact. These results show that inhibition of the chaperone function of Hsp90 via geldanamycin decreased the association of the assessed viral proteins with Hsp90.

### Inhibition of the proteasome pathway decreases viral protein degradation.

**FIG 5:**
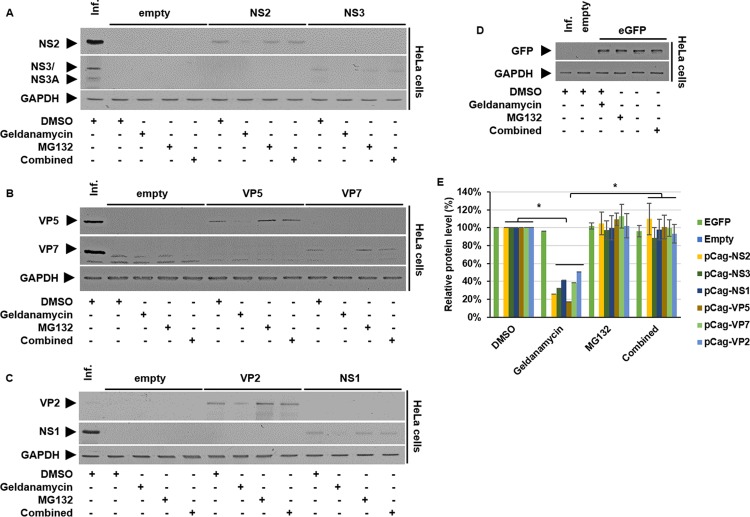
Inhibition of Hsp90 activity facilitates the proteasome-mediated turnover of viral proteins. HeLa cells were transiently transfected with the indicated plasmids for 24 h before being treated with DMSO, 300 nM geldanamycin, 250 nM MG132, or geldanamycin and MG132 combined for 24 h. (A to D) Samples were harvested 48 hpt and analyzed by Western blotting with specific antibodies as indicated. (E) Densitometry analysis results for the Western blots are expressed as percentages, normalized to GAPDH. Error bars represent the SD values of stimulations from three independent experiments. An asterisk (*) denotes a significant difference from the control (*P* < 0.05).

To investigate further the direct influence of Hsp90 on BTV proteins in the absence of virus replication, we examined the transient expression of individual structural and nonstructural virus proteins. We first assessed the chaperone function of Hsp90 to determine whether the decrease in the expression of the viral proteins was due to proteasomal degradation via the ubiquitin-proteasome pathway. HeLa cells were transfected with either a plasmid expressing a viral protein (NS1, NS2, NS3, VP2, VP5, and VP7) or a enhanced green fluorescent protein (eGFP)-control plasmid for 24 h and subsequently treated with DMSO, geldanamycin (300 nM), the proteasome inhibitor MG132 (250 nM), or geldanamycin and MG132 combined for a further 24 h. Cell lysates were then analyzed by Western blotting with specific antibodies for each protein (Fig. 5A to D). As shown in Fig. 4E, geldanamycin treatment decreased protein levels between 50 and 80%, specifically, NS1 by ∼60% ± 13%, NS2 by ∼75% ± 13%, NS3 by ∼70% ± 10%, VP2 by ∼50% ± 14%, VP5 by ∼80% ± 7%, and VP7 by ∼60% ± 13%. These decreases correlate with the decreases observed during a viral infection (Fig. 1B). There was no significant change in protein levels associated with the proteasomal inhibitor MG132 treatment. However, in the presence of geldanamycin and the proteasomal inhibitor during the combined treatment, viral protein levels were restored, showing a significant recovery from geldanamycin-only treatment levels. These data confirmed a role for Hsp90 in stabilizing these viral proteins, preventing their turnover via the proteasome pathway. Further, this effect appeared to be specific for the assayed viral proteins, since the protein levels of the transfected eGFP-control showed no significant change during the same treatment regime.

### In contrast to Hsp90 inhibition, inhibition of Hsp70 does not affect BTV protein levels or virus titer.

**FIG 6:**
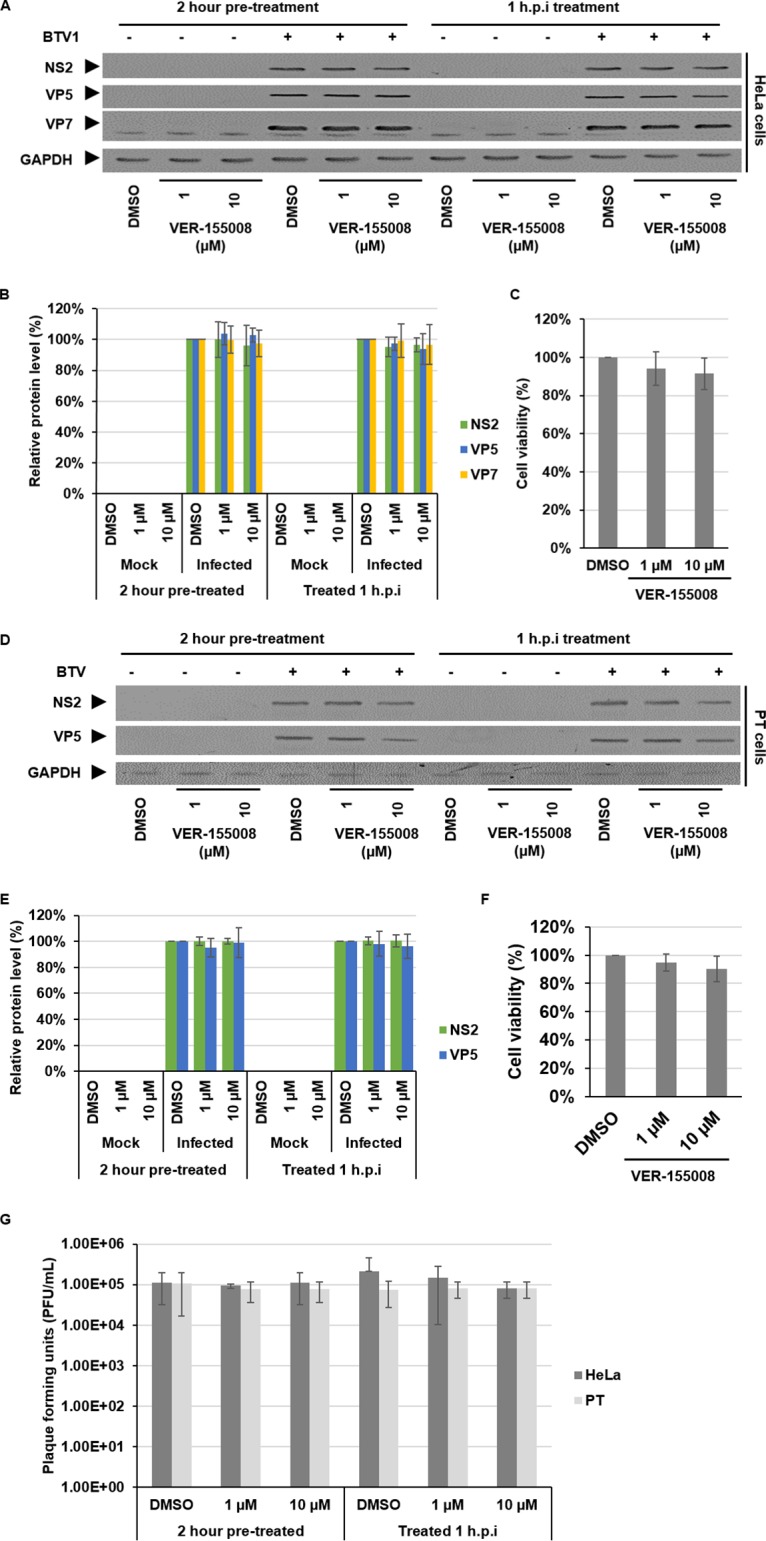
Inhibition of Hsp70 prior to or after infection does not affect BTV protein levels. HeLa cells (A to C) and PT cells (D to F) were treated with DMSO or 1 or 10 μM VER-155008 for 2 h prior to or at 1 hpi with BTV1 (MOI = 1) and harvested 18 hpi. As controls, mock-infected and DMSO-treated cells were included. (A and D) Samples were analyzed by Western blotting with specific antibodies as indicated. (B and E) Densitometry analysis results for Western blots are expressed as percentages, normalized to GAPDH. (C and F) Viability of cells treated with DMSO or 1 or 10 μM VER-155008 for 24 h. (G) Virus titers (PFU/ml) derived from HeLa and PT cells infected with BTV1 (MOI = 1) and treated as described above. Error bars represent the SD values of stimulations from three independent experiments. An asterisk (*) denotes a significant difference from the control (*P* < 0.05).

**FIG 7:**
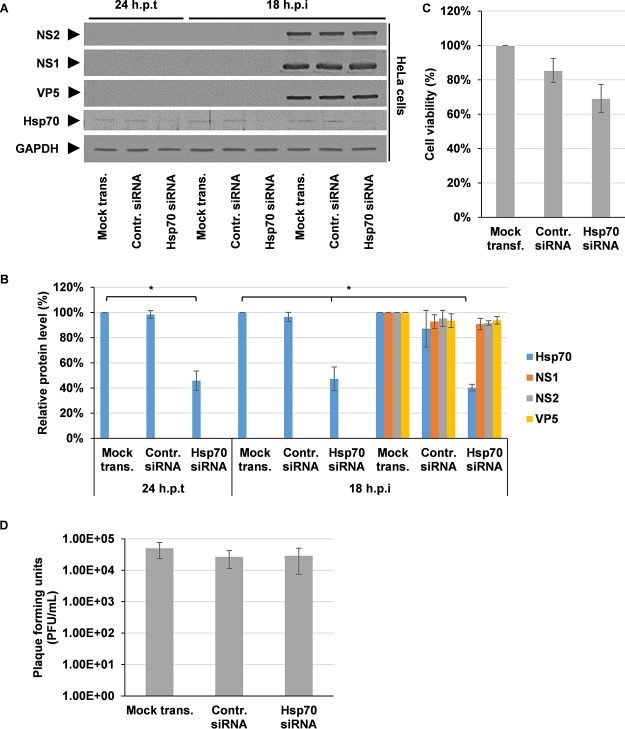
siRNA knockdown of Hsp70 does not affect BTV protein levels. HeLa cells were either mock transfected or transfected with 400 nM control siRNA or Hsp70 siRNA for 24 h. Transfected cells were then infected with BTV1 (MOI = 1) for 18 h. (A) Samples were analyzed by Western blotting with specific antibodies as indicated. (B) Densitometry analysis results for Western blots are expressed as percentages representing NS1, NS2, VP5, and Hsp70 protein levels, normalized to GAPDH. (C) Viability of cells transfected with mock, negative-control siRNA and Hsp70 siRNA for 42 h. (D) Virus titers (PFU/ml) derived from HeLa cells 18 hpi. Error bars represent the SD values of stimulations from three independent experiments. An asterisk (*) denotes a significant difference from the control (*P* < 0.05).

Hsp90 can function in concert with Hsp70 via the cochaperone Hsp70/Hsp90 organizing protein (HOP). This creates an active complex in which client proteins are transferred from Hsp70 to Hsp90 to advance their folding (26, 27). Here, we assessed what effect the inhibition of Hsp70 had on BTV replication. To inhibit Hsp70 function, we used a specific inhibitor of Hsp70, VER-155008 (28). HeLa cells were either pretreated 2 h prior to infection or at 1 hpi with either DMSO or 1 or 10 μM VER-155008. Under both conditions, inhibition of Hsp70 did not result in any significant changes, neither increasing nor decreasing the levels of structural protein (VP5 and VP7) or the level of the nonstructural protein (NS2) at the indicated concentrations (Fig. 6A and B). To examine whether the inhibitor concentrations used resulted in cellular toxicity, the effect of the inhibitor on cell viability was assessed by an MTT assay. The data showed that 1 or 10 μM VER-155008 was not toxic to HeLa cells (Fig. 6C). Furthermore, we did not observe any effect on virus titers, which neither increased nor decreased (Fig. 6G). Replicate experiments using sheep PT cells confirmed the observations made using HeLa cells. Analysis of viral protein levels showed no significant increases or decreases (Fig. 6D and E). Cell viability was also not found to be compromised (Fig. 6F). Furthermore, there was no increase or decrease in virus titer evident (Fig. 6G). Cumulatively, these data indicate that Hsp70 inhibition in HeLa and sheep PT cells does not affect BTV protein levels or virus titer, mediating neither increases nor decreases, independent of inhibition prior to or after infection. To mitigate the possibility of off-target effects or that the inhibitor concentrations used were insufficient to affect BTV, we undertook siRNA knockdown experiments targeting Hsp70. HeLa cells were mock transfected or transfected either with a control siRNA or with Hsp70 siRNA for 24 h prior to infection. At 24 hpt, the samples were assessed to confirm the successful knockdown of Hsp70 protein levels prior to infection. The cells were then infected with BTV, harvested 18 hpi, and analyzed by Western blotting (Fig. 7A). Densitometry quantification confirmed that at the time of infection (24 hpt) the Hsp70 protein levels normalized to GAPDH had decreased by ∼54% ± 8%. Upon infection, we observed no increase or decrease in NS1, NS2, or VP5 protein levels that correlated to the corresponding decreased levels of Hsp70 protein. The level Hsp70 protein at 18 hpi was decreased by ∼60% ± 3% (Fig. 7B). To address whether the siRNA knockdown of Hsp70 resulted in cellular toxicity, cell viability was assessed by an MTT assay. The data showed that HeLa cell viability decreased ∼30% ± 8% using the Hsp70 siRNA; however, this decrease was comparable to the decrease observed using the negative-control siRNA, which decreased cell viability by ∼15% ± 7% (Fig. 7C). Furthermore, when the supernatants from the Hsp70 siRNA knockdown samples were analyzed, we observed no increase or decrease in virus titers (Fig. 7D). Cumulatively, the siRNA knockdown of Hsp70 correlates with the data using the inhibitor VER-155008, showing no significant increases or decreases in BTV protein levels or virus titers.

## DISCUSSION

To date, viral reliance upon Hsp90 appears pervasive, with viral replication showing a hypersensitivity to Hsp90 inhibition at concentrations that do not compromise cellular viability (12). This hypersensitivity may derive from unique aspects of the viral proteome and the burden that it places upon the host-cell protein folding machinery. First, the viral proteins may be structurally complex, which entails a susceptibility to misfolding and aggregation, requiring extensive protein stabilization. Second, the proteins are synthesized in copious quantities during a brief period of time that generates a high demand for chaperones (12, 29–32). These aspects may combine and create a vulnerability to even minor perturbations to the host cell protein folding capacity. Furthermore, specific examples show how certain RNA viruses, such as human immunodeficiency virus type 1 (33), Ebola virus (34), vesicular stomatitis virus (35), poliovirus (31), mumps virus (36), and hepatitis C virus (37) exhibit a dependency on Hsp90 to facilitate transcription and cell survival, virus propagation, protein folding, and replication complex formation. Thus far, for members of the *Reoviridae*, it has been reported that Hsp90 facilitates dimerization of nonstructural protein 3 (NSP3) of rotavirus (38) and participates in the biogenesis of trimeric cellular attachment protein sigma1 of reovirus (39).

During the Hsp90 inhibition experiments with geldanamycin, we found that inhibition prior to virus entry did not significantly impair virus replication beyond the significant decreases that were observed during inhibition postinfection. This signified that Hsp90 may not have played a role during virus entry versus that reported for dengue virus (40). Furthermore, the significant decrease in viral protein levels and titers could also be recapitulated in a natural host-derived cell line, the sheep PT cells. For both cell lines, the effective concentration of geldanamycin did not significantly influence cell viability, as measured by an MTT assay, while proving detrimental to BTV replication. This is significant for showcasing the possibility of a therapeutic intervention using geldanamycin in a natural host of BTV.

A time-course experiment examining viral and host cell protein levels, viral dsRNA synthesis, and virus titers at 4, 8, and 18 hpi showed that profile differences already existed at 4 hpi. Although protein levels at 4 hpi were significantly lower compared to the DMSO control, the viral genomic copy numbers had not significantly decreased. This indicated that the low abundance of viral protein present in the geldanamycin-treated samples was not due to a lack in viral genomes that could function as templates for viral mRNA synthesis. Furthermore, as the infection progressed, at 8 hpi, in addition to decreased viral protein levels, viral genome copy numbers were also decreased compared to the DMSO control. This could indicate that a lack of sufficient viral proteins to drive virus replication resulted in the discrepancy in viral genome copy numbers. At 18 hpi, as observed at 8 hpi, both viral protein levels and genome copy numbers were significantly lower compared to the DMSO control, with the genome copy numbers having decreased further relative to the DMSO control. Overall, the lack of viral proteins and viral genomes was reflected in the decreased viral titers that were observed at these latter time points. Furthermore, at all assayed time points, CK2α protein levels remained unperturbed, suggesting that inhibition of Hsp90 did not affect an essential host protein for BTV replication.

During the Hsp70 inhibition experiments with VER-155008, we could not detect any significant effect on BTV replication. Inhibition of Hsp70 did not significantly decrease or increase viral protein levels (structural and nonstructural) or titers in both HeLa and sheep PT cells. This clearly separates BTV from rotavirus, in which it has previously been reported that inhibition of Hsp70 mediated increases in structural protein levels and virus titers (41). In regard to cell viability, in both cell lines the concentrations of VER-155008 that were used had no significant effect. Furthermore, the observed decreases in cell viability, as measured by the MTT assays, for siRNA knockdowns of Hsp70 and Hsp90 could be due to a cellular antiproliferative response, which has been previously observed for Hsp70 and Hsp90 siRNA knockdowns (42, 43). However, this effect was compensated for by the equal loading of protein samples during Western blotting.

Mechanistically, while it has been reported that the active HOP/Hsp70/Hsp90 complex facilitates client protein folding (26, 27), both Hsp70 and Hsp90, in conjunction with the cochaperone carboxyl terminus of Hsc70-interacting protein (CHIP), can facilitate the ubiquitination of defective client proteins for proteasomal degradation (44). Our data showed that Hsp90 could coimmunoprecipitate the viral proteins NS1, NS2, NS3, VP2, VP5, and VP7 and the host cell proteins Hsp70 and CK2α. The presence of Hsp70 and CK2α indicated that the cochaperone protein complexes formed with Hsp90 remained intact and unperturbed. Further, when singly expressing these proteins, it proved possible to show that the chaperone function of Hsp90 prevented the viral proteins from undergoing proteasomal degradation, since blocking proteasomal function using MG132 in the presence of geldanamycin restored viral protein levels. This shows that the Hsp90 chaperoning of viral proteins and preventing their degradation via the proteasome is a mechanism of action exploited by BTV. Furthermore, that Hsp70 inhibition did not influence BTV protein levels suggests that it was not as essential as Hsp90 for preventing the proteasomal degradation of BTV proteins.

Cumulatively, our data show that during a BTV infection Hsp90 performs in its canonical capacity, functioning to chaperone and stabilize viral proteins, safeguarding them from proteasomal degradation. Inhibition of Hsp90 did not result in decreased levels of the host cell CK2α protein, which is an Hsp90 interactor and client (24, 25) and is also essential for BTV replication (20). This indicated that the effect of inhibition of Hsp90 activity on BTV replication was not an off-target effect due to depriving BTV of CK2α. Furthermore, these data suggest that throughout the infection, inhibition of Hsp90 resulted in the proteasomal degradation of viral proteins, which led to an inability to efficiently replicate viral genomes and produce infectious virus particles. As had been observed with Hsp90, a similar function could not be confirmed for Hsp70, as viral protein levels remained unperturbed by either pharmacological intervention or siRNA knockdown of Hsp70. In conclusion, our study demonstrates that Hsp90 may be listed among the expanding pantheon of host factors coopted by BTV to facilitate virus replication.

## MATERIALS AND METHODS

### Cell lines and virus.

BSR cells (BHK-21 subclone, BHK21 cells (ATCC CCL10), HeLa cells (ATCC CCL-2), and sheep PT cells (ovine-derived kidney cells, ATCC CRL-1633) were maintained in Dulbecco modified Eagle medium (Sigma-Aldrich Co.). The medium was supplemented with 10% (vol/vol) fetal bovine serum (FBS; Invitrogen), 100 U of penicillin/ml and 100 μg of streptomycin/ml (Sigma-Aldrich Co.), and minimal essential medium nonessential amino acids (Gibco). BTV serotype 1 (BTV-1) stock was obtained by infecting BSR cells at a low MOI and harvested when a 100% cytopathic effect was evident. Virus stocks were stored at 4°C.

### Plasmids.

Transient protein expression plasmids were previously generated by cloning the open reading frames downstream of the chicken β-actin promoter of the pCAGGS expression vector reported previously (45, 46) to generate pCAG VP2, pCAG VP5, pCAG VP7, pCAG NS1, pCAG NS2, pCAG NS3, and eGFP.

### Pharmacological reagents.

The HSP90 inhibitor geldanamycin was purchased from InvivoGen, and the HSP70 inhibitor VER-155008 was purchased from Sigma-Aldrich Co. All reagents were used at the concentrations specified.

### Immunofluorescence microscopy.

HeLa cells were grown on glass coverslips to 90% confluence prior to infection with BTV1 (MOI = 1). At specified times, the cells were washed with phosphate-buffered saline (PBS) before being fixed for 10 min in 4% paraformaldehyde. Cells were permeabilized and blocked using 1% bovine serum albumin (BSA) in 0.1% PBS-Tween for 1 h to permeabilized the cells and block nonspecific protein-protein interactions. The cells were then incubated with the primary antibody primary antibody NS2 (guinea pig anti-NS2 serum) for 1 h at room temperature. The cells were washed in PBS before incubation with secondary antibody [goat anti-guinea pig IgG(H+L) secondary antibody], Alexa Fluor 488 conjugate-A-11073 [Thermo Fisher]), and Hoechst 33342 (Invitrogen) for 1 h at room temperature. The cells were washed with PBS before being mounted on slides on mounting medium (Invitrogen).

### siRNA knockdowns.

HeLa cells were transfected with 400 nM Silencer Negative Control No. 1 siRNA (AM4611; Ambion), with HSP90AB1 Silencer validated siRNA (AM51331; Ambion), or with Hsp701A Silencer select predesigned siRNA (s194536; Thermo Fisher) using the Lipofectamine RNAiMAX transfection reagent (13778-100; Invitrogen). Transfections were carried out according to the supplier’s instructions. Cells were transfected for 24 h prior to BTV1 infection (MOI = 1). Samples were harvested at the indicated time points for Western blot analysis or plaque assay.

### Western blot analysis.

SDS-PAGE gels were transferred via a semidry blotter to polyvinylidene difluoride transfer membranes and blocked for 4 h with TBS-T containing 10% (wt/vol) milk powder. Primary antibodies were used to detect Hsp90 (rabbit anti-HSP90AB1 [11405-1-AP; Proteintech]), Hsp70 (mouse anti-Hsp70 [ab2787]), CK2a (rabbit anti-CSNK2A1 [ab10466]), NS2 (guinea pig anti-NS2 serum), VP2 (rabbit anti-VP2 serum), VP5 (guinea pig anti-VP5 serum), VP7 (guinea pig anti-VP7 serum), NS1 (rabbit anti-NS1 serum), NS3 (rabbit anti-NS3 serum), GFP (mouse anti-GFP [G1546-200UL]; Sigma-Aldrich Co), and GAPDH (rabbit anti-GAPDH (ab9485; Abcam). These were added to blocked membranes and incubated overnight at 4°C. Secondary antibodies included an alkaline phosphatase-conjugated goat anti-guinea pig immunoglobulin G (1:10,000; Sigma-Aldrich Co., A5062), goat anti-rabbit (A0418), and an alkaline phosphatase-conjugated goat anti-mouse (A3562) IgG (1:10,000; Sigma-Aldrich Co.), respectively.

### Coimmunoprecipitation assay.

HeLa cells were grown in 10-cm tissue culture dishes. Mock infections and infections were carried out according to a standard protocol. At 17 hpi, the cells were treated with DMSO or 300 nM geldanamycin for 1 h prior to harvesting. Cell lysates (1 ml) were incubated with 100 μl of a protein A-Sepharose bead slurry (P3391; Sigma-Aldrich) conjugated to isotype control (rabbit anti-FLAG [F7425; Sigma-Aldrich]) or Hsp90 antibody (rabbit anti-HSP90AB1 [11405-1-AP; Proteintech]). Lysate and beads were gently mixed overnight on ice. Subsequently, the mixture was centrifuged at 2,000 × *g* for 2 min at 4°C, and the supernatant was discarded. The beads were washed four times with lysis buffer. The beads were resuspended in 50 μl of 2× SDS sample buffer and heated at 100°C for 5 min, followed by centrifugation at 2,000 × *g* for 3 min. The proteins were separated by electrophoresis through SDS-PAGE gels, and Western blot analysis was carried out as described above.

### MTT assay.

The cell viability of HeLa and PT cells was measured by using an MTT assay (Sigma). In brief, HeLa and PT cells were seeded in 96-well plates at a density of 10^4^ cells per well in 100 μl of medium. The plates were incubated in a 37°C humidified incubator for adherence overnight. The cells were then treated with the indicated inhibitor (24 h) or transfected with the indicted siRNA (42 h). The formazan dye was detectable by spectrophotometric analysis (*A*_540_; Spectramax plate reader).

### Plaque assays.

HeLa cells were grown in 12-well plates. Cells were either pretreated for 2 or 1 h postinfection with 300 nM geldanamycin or 1 or 10 μM VER-155008 or DMSO control. Cells were infected with BTV1 or the supernatant of previously harvested cells. After adsorption for 30 min at 4°C, the cells were incubated at 37°C in growth medium for 1 h in the presence of inhibitor. The growth medium was removed and replaced with 0.6% Avicel (FMC BioPolymer) overlay medium (Eagle minimal essential medium containing l-glutamine, 10% FBS, and antibiotics) in conjunction with inhibitors, where appropriate. Cells were incubated at 37°C for 72 h before being fixed with 4% paraformaldehyde and subsequently stained with crystal violet. Titers are expressed as PFU/ml.

### Focus-forming assay.

HeLa cells were grown in 12-well plates. Cells were either pretreated for 2 h or at 1 h postinfection with 300 nM geldanamycin or DMSO control. The cells were infected with BTV1. After adsorption for 30 min at 4°C, the cells were incubated at 37°C in growth medium for 1 h in the presence of inhibitor. Growth medium was removed and replaced with fresh medium containing either 300 nM geldanamycin or DMSO control. The cells were incubated at 37°C for 18 h before being fixed with 4% paraformaldehyde. The cells were permeabilized with ice-cold methanol for 10 min, washed with PBS, and then blocked with 1% BSA in PBS for 1 h at room temperature. The cells were incubated with the primary antibody NS2 (guinea pig anti-NS2 serum) for 4 h at room temperature and then washed in PBS before being incubated with secondary antibody alkaline phosphatase-conjugated goat anti-guinea pig IgG (1:10,000; Sigma-Aldrich Co., A5062) for 1 h at room temperature. The cells were then washed with PBS before incubation with BCIP/NBT chromogenic substrate.

### qRT-PCR.

For the detection of genomic single-stranded RNA, BTV-1 segment 6 were analyzed by qRT-PCR using the primers reported by Toussaint et al. (47). BTV viral RNAs from the experiments were reverse transcribed with segment 6 forward primer (GGCAACYACCAAACATGGA) into cDNA using ReverseAid premium reverse transcriptase (Thermo) and quantified with suitable primers using the 7500 Fast Real-Time PCR system and SYBR select master mix (Applied Biosystems). Three independent experiments were undertaken, and qPCR was performed in duplicate. Standard deviations (SD) from the three experiments were calculated.
